# Adsorption of Indigo Carmine Dye by Acacia nilotica sawdust activated carbon in fixed bed column

**DOI:** 10.1038/s41598-022-19595-6

**Published:** 2022-09-15

**Authors:** Tripti Gupta, Khalid Ansari, Dilip Lataye, Mahendra Kadu, Mohammad Amir Khan, Nabisab Mujawar Mubarak, Rishav Garg, Rama Rao Karri

**Affiliations:** 1 Department of Civil Engineering, Shri Ramdeobaba College of Engineering and Management, Nagpur, 440013 India; 2 Department of Civil Engineering, Yeshwantrao Chavan College of Engineering, Nagpur, 441110 India; 3 Department of Civil Engineering, Visvesvaraya National Institute of Technology, Nagpur, 440010 India; 4 Department of Civil Engineering, Galgotia College of Engineering, Knowledge Park I, Greater Noida, Uttar Pradesh 201310 India; 5 Petroleum and Chemical Engineering, Faculty of Engineering, Universiti Teknologi Brunei, Bandar Seri Begawan, BE1410 Brunei Darussalam

**Keywords:** Environmental sciences, Environmental social sciences, Chemistry, Energy science and technology, Materials science

## Abstract

A continuous mode fixed-bed up-flow column adsorption analysis was conducted utilizing *Acacia nilotica* sawdust activated carbon (ASAC) as an adsorbent for the adsorption treatment of toxic Indigo Carmine Dye (ICD). The effect on the adsorption characteristics of ASAC of the influent ICD concentration, flow rate, and column bed depth has been investigated. According to the column study, the highest efficiency of ICD removal was approximately 79.01% at a preliminary concentration of 100 mg/L with a flow rate of 250 mL/h at a bed depth of 30 cm and adsorption power of 24.67 mg/g. The experimental work confirmed the dependency of break-through curves on dye concentration and flow rate for a given bed depth. Kinetic models were implemented by Thomas, Yoon–Nelson, and Bed-depth-service-time analysis along with error analysis to interpret experimental data for bed depth of 15 cm and 30 cm, ICD concentration of 100 mg/L and 200 mg/L and flow rate of 250 mL/h, and 500 mL/h. The analysis predicted the breakthrough curves using a regression basin. It indicated that all three models were comparable for the entire break-through curve depiction. The characteristic parameters determined by process design and error analysis revealed that the Thomas model was better followed by the BDST and Yoon–Nelson models in relating the procedure of ICD adsorption onto ASAC. B-E-T surface area and B-E-T pore volume of ASAC were 737.76 m^2^/g and 0.2583 cm^3^/g, respectively. S-E-M and X-R-D analysis reveal the micro-porous and amorphous nature of ASAC. F-T-I-R spectroscope indicate distinctive functional assemblies like -OH group, C–H bond, C–C bond, C–OH, and C–O groups on ASAC. It could be computed that the ASAC can be used efficiently as an alternative option for industrial wastewater treatment

## Introduction

Colorants and dyestuffs are commonly used in manufacturing and commercial industries, including clothing, rubber, pharmacy, leather, printing press, fruit, cosmetics, carpet, and paper. The textile industry ingests more than 80% of the entire production of dyestuff, creating it the principal consumer ^1^. About 10–15% of textile industry dyes are discharged in streams, making the effluents aesthetically unpleasant. Discharge of such colored effluents is dangerous from an environmental and ecological point of view. Color obstructs sunlight dispersion, hinders photosynthesis action, and constrains the growth and metabolism of aquatic biota. The eradication of color from the effluent-carrying dye is a crucial challenge owing to difficulties in handling conventional and fixed treatment methods to manage such wastewaters. Consequently, such techniques are ineffective but cannot be utilized to handle the large variety of organic pigment discharge efficiently.

Surface assimilation, or the deposit of impurities on the surface of a solid, is an attractive alternative treatment. Suitable for its convenience, simplicity of use, handling, sludge-free facility, and rejuvenation potential, it has become trendy and appealing, demonstrating an appropriate process for extracting non-biodegradable chemicals (specifical dyes) from wastewater^2^. Industrial activated carbon (AC) is a well-known adsorbent used for admirable adsorption capabilities. However, it is expensive, and its rejuvenation makes it pricier in some world regions (e.g., Asia). Hence it is desirable to search for low-cost alternatives such as natural ingredients, agricultural by-products, or industrial waste as an adsorbent material. These products do not need any additional or expensive pre-treatment and should be regarded as possible adsorbents to eliminate dye-containing wastewater. These low-cost products provide acceptable output for diagnosing-colored effluents in laboratory measurements^3^.

Several papers reported that manylow-cost materials such as natural, agricultural, or industrial wastes like Acacia nilotica sawdust activated carbon^4^, orange peels activated carbon^5^, chicken feathers^6^, pongamia pinnata seed shell activated carbon^7^, palm wood cellulose activated carbon^8^,banana peels activated carbon^9^, acacia glauca sawdust activated carbon^10^, babul sawdust activated carbon^11^, etc. have been considered to adsorb dyestuffs, heavy metals, and other impurities from solution as unconventional adsorbents. Mall et al. ^12,13^ provided a critical analysis of such minimum cost adsorbents to diagnose different wastewaters to remove several toxins carrying wastewaters. Sorption of different adsorbates using other adsorbent materials like boron by sepiolite ^14^, azo dye by jute fibers^15^, phenolic compounds^16^, methylene blue dye by zeolite ^17^, etc. in column mode is also described by some researchers. But dye adsorption (specifically Indigo Carmine Dye) by activated carbon of *Acacia nilotica* sawdust in column mode is hardly reported. Indigo Carmine is one of the dark blues colored poisonous and toxic, crystalline type of powdered dye having a chemical composition of C_16_H_8_Na_2_O_8_S_2_N_2_, molecular weight is 466.367 g/mol, and distinctive wavelength of 610 nm. It is very commonly used as a colorant and an indicator of pH in various activities. It has some drug allergies due to which it can damage the life of man ^18^, affects bones and chromosomes, and can cause dangerous hemodynamic effects on living beings ^19^. This study investigates the efficacy of Acacia nilotica sawdust activated carbon (ASAC) foradsorptive elimination of poisonous IndigoCarmine Dye (ICD) in a constant stable bed up-flow column.

The current study used a perspex column for continuous fixed-bed up-flow column analysis. The adsorbent of known weight for a given bed depth, i.e., *Acacia nilotica* sawdust activated carbon (ASAC), was packed with glass beads from the top and bottom. The adsorbate, Indigo Carmine Dye solution (ICD), was pumped at the appropriate flow rates using a pump with a known initial concentration at natural pH. The final samples were taken at daily interims at the column's output, along with the concentrations were determined using a spectrophotometer.

## Materials and methods

The objective of the present research work is to utilize *Acacia nilotica* sawdust activated carbon (ASAC) as an adsorbent for the adsorptive treatment of toxic Indigo Carmine Dye (ICD) bearing wastewater. For this purpose, continuous mode fixed-bed up-flow column adsorption analysis is conducted. The effect of the adsorption characteristics of ASAC on the influent ICD concentration, flow rate, and column bed depth has been investigated.

### Adsorbent preparation and adsorbent characterization (ASAC)

For continuous fixed-bed up-flow column study, the adsorbent material,i.e., carbon activated babool sawdust for the removal of ICD, was prepared by chemically activating the material with ortho-phosphoric acid. The plant chosen for the study is the Acacia nilotica tree. Acacia nilotica is a scientific name for the evergreen Babool tree. It is native to Africa, the Middle East, the Indian subcontinent and across Asia. It is locally available and found abundantly. This plant is not directly utilized as an adsorbent in the present research work. The sawdust of babool tree which is a waste material from sawmills and hence of marginal cost has been considered here to prepare the adsorbent. Sawmill and timber industries are commonly available sources of sawdust or wood waste. Secondly the plant is locally available and found abundantly across Asia. In addition to this, Acacia nilotica tree/sawdust/wood waste is not listed as vulnerable/rare/endangered/indeterminate. The cost-effective low cost raw sawdust material, obtained from the local saw mill (M/s Gopal Timber Mill) in a quantity of 0.5 kg, was crushed and sieved according to the protocol outlined in Part 4 of Bureau of Indian Standards IS-2720^20^ to get a uniform size in the range of 250–500 μ. It is then rinsed with doubly distillate water, naturally dehydrated, and incubated in an oven at 105 °C for around 2 h. After this, char is obtained by mixing 25 mL of ortho-phosphoric acid, i.e., H_3_PO_4_, in 50 g dehydrated sawdust in a 0.5:1 volume to weight ratio. To complete the activation and carbonization, the char was placed in a muffle furnace for around 1 h at 450 °C. The carbon was then rinsed with doubly distillate water for 2 h, dried at 378 K, and used in the new adsorption column analysis as *Acacia nilotica* sawdust activated carbon (ASAC)^4^.

Characterization of adsorbent ASAC includes Brunauer, Emmett and Teller (*B-E-T*) surface area and (*B-E-T*) pore volume analysis, scanning electron microscope (*S-E-M*) analysis, Fourier transform infrared (*F-T-I-R*), spectroscopy, and X-ray diffraction (*X-R-*D) technique. Adsorption is a surface process. It robustly depends on the adsorbent’s surface characteristics. The area-volume, morphology, chemistry and constitution of the ASAC surface were premeditated by *B-E-T*, *S-E-M*, *F-T-I-R*, and *X-R-D* analysis.

### Adsorbate (ICD) preparation

Analytical reagents were used in the current research. Indigo Carmine Dye (ICD) was acquired from a scientific store-Upper India, Nagpur. Standard 1000 mg/L stock solution was obtained by dissolving 1 g powdered ICD in 1000 mL doubly distillate water. Dilution of a standard stock of 1000 mg/L concentration yielded desired solutions of 100 mg/L and 200 mg/L concentrations. A double-beam Shimadzu ultraviolet–visible spectrophotometer was used to calculate the wavelength at an absorbance of 610 nm. (Model No. 2450).

### Column study

**Figure 1:**
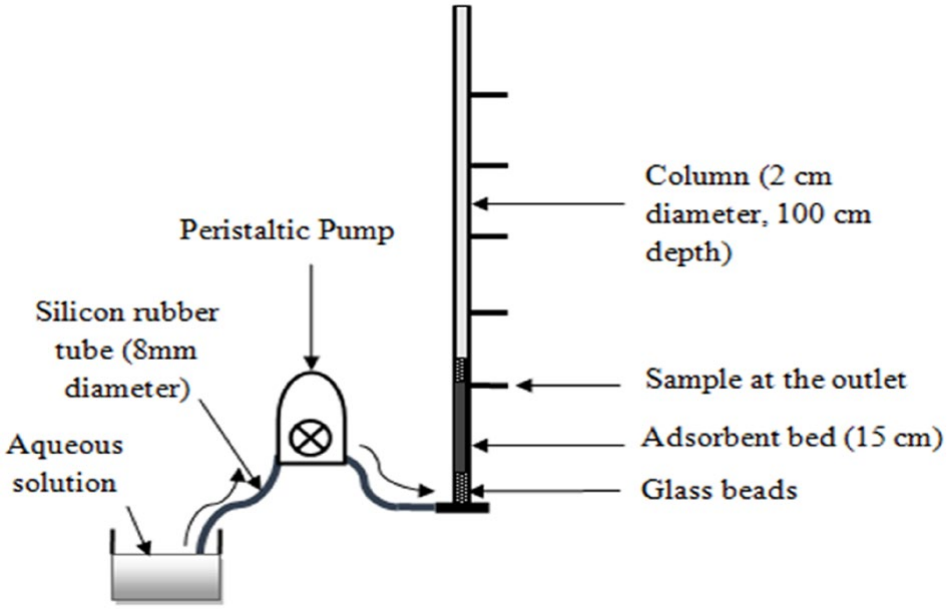
Experimental setup of column study.

Continuous flow analysis fully explores the concentration differential, which is believed to be a prime factor for adsorption, resulting in more optimal use of the adsorbent potential and improved effluent performance ^21^. The schematic diagram showing the set-up of the column study is shown in Fig. 1.

### ICD adsorption in the fixed bed system

The fixed bed adsorption analysis was performed using perspex glass columns and consists of internal diameters of 2 cm and lengths of 100 cm. The experiments were conducted using doubly distillate water at natural flow and continued till the bed reached the exhaustion point of all the pH of respective solutions. Table 1 shows the operating conditions for each set.

**Table 1:** Operational conditional for ASAC in fixed bed study.

Effect of study	Column bed depth (cm)	Influent dye concentration (mg/L)	Flow rate (mL/h)
Influent dye concentration	15	100	250
		200	
	15	100	500
		200	
	30	100	250
		200	
	30	100	500
		200	
Flow rate	15	100	250
			500
	15	200	250
			500
	30	100	250
			500
	30	200	250
			500
Column bed depth	15	100	250
	30		
	15	200	250
	30		
	15	100	500
	30		
	15	200	500
	30		

The predetermined flow rates of 250–500 mL/h, the predetermined ICD concentration of 100–200 mg/L and the predetermined column bed depth of 15–30 cm were adopted in the present research work in order to examine the effect on the adsorption performance and to establish optimum conditions for the adsorption of ICD in a column by ASAC.

## Results and discussion

Continuous flow analysis is a successful periodic desorption mechanism. The breakthrough curve is the efficiency of a continual adsorption sample or a fixed-bed column. A break-through curve happens if the outflow agglomeration from a column bed is (3–5) % of the inflow agglomeration^22^. The breakthrough curve is drawn based on lapse time by plotting output concentration against initial concentration, flow rate, bed width, and column diameter. In a fixed bed study, the adsorbent closest to the contaminated water saturates first, where maximum adsorption occurs initially. As time passes, these adsorption areas advance until they enter the bed exit ^23^. As the adsorption areas migrate through the column, the adsorbate concentration equals the feed concentration at the exit.

### Impact of ICD concentration on ASAC break-through plots

Break-through time (t_b_) effect on initial concentration on ICD adsorption on the ASAC's fixed-bed column was investigated, and measurements were conceded out at the preliminary stage of 100–200 mg/L concentration with a maintained flow discharge of 250 mL/h and 500 mL/h with column bed depths of 15 cm and 30 cm.The obtained results are depicted in Table 2.

**Table 2:** The effects of preliminary ICD concentration on the ASAC break-through curve.

Columnbed depth (cm)	Flowrate (mL/h)	Concentration (mg/L)	Break-through time tb (min)	Exhaust time text (min)	Percentage removal (%)	Adsorption capacity (mg/g)
15	250	100	240	1350	74.48	25.13
		200	90	660	57.35	23.43
30	250	100	570	2670	79.01	24.67
		200	240	1320	65.79	22.45
15	500	100	120	810	68.38	25.69
		200	45	570	47.35	21.99
30	500	100	270	1560	75.50	21.24
		200	120	1290	58.43	21.05

**Figure 2:**
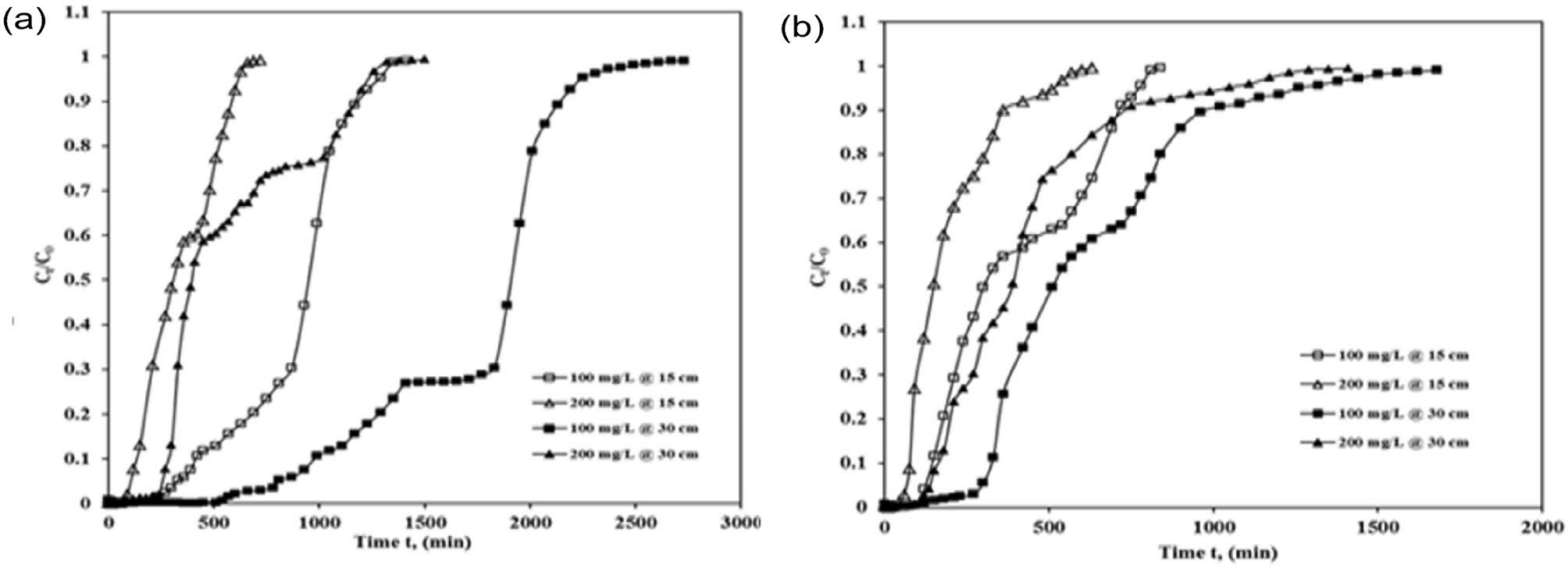
Break-through curve showing effect of concentration of ICD sorption by ASAC (z = 15 cm and 30 cm, C_0_ = 100 mg/L and 200 mg/L, at (**a**) Q = 250 mL/h and (**b**) Q = 500 mL/h).

It was observed that break-through time generally occurred faster by rising in preliminary ICD concentration. The break-through time and exhaust time of ASAC were observed to increase with the decrease in initial ICD concentration, whereas the percent removal and adsorption capacity reduced with an increase in initial ICD concentration. ASAC's adsorption capacity decreases, and column percentage removal capacity also decreases because of the inadequate residence time of the column’s dye solution. For bed depth of 15 cm and the constant flow of 250 mL/h, the break-through and exhaust times for 100 mg/L and 200 mg/L were 240 min, 90 min and 1350 min, and 660 min, respectively. Likewise, for the constant flow of 250 mL/h, the percentage removal and the adsorption capacity for 100 mg/L and 200 mg/L were found to be 74.48%, 57.35% and 25.12 mg/g, 23.43 mg/g, respectively (as shown in Table 2 and Fig. 2a).

Similarly, in the case of a depth of 30 cm and the constant flow of 250 mL/h, the break-through time and exhaust time for 100 mg/L and 200 mg/L were 570 min, 240 min, 2670 min, and 1320 min, respectively. Likewise, for the constant flow of 250 mL/h, the percentage removal and the adsorption capacity for 100 mg/L and 200 mg/L were found to be 79.01%, 65.79% and 24.67 mg/g, 22.45 mg/g, respectively (as shown in Table 2 and Fig. 2a).

Similar trends were observed for the depth of 15 cm and constant flow of 500 mL/h, for which the break-through time and exhaust time for 100 mg/L and 200 mg/L were found to be 120 min, 45 min, 810 min, and 570 min, respectively. Likewise, for the constant flow of 500 mL/h, the percentage removal and the adsorption capacity for 100 mg/L and 200 mg/L were found to be 68.38%, 47.35%, and 25.69 mg/g, 21.99 mg/g, respectively (as shown in Table 2 and Fig. 2b). Again, similar trends were observed for thedepth of 30 cm constant flow of 500 mL/h, for which the break-through time and exhaust time for 100 mg/L and 200 mg/L were found to be 270 min, 120 min, 1560 min, 1290 min, respectively. Likewise, for the constant flow of 500 mL/h, the percentage removal and the adsorption capacity for 100 mg/L and 200 mg/L were found to be 75.50%, 58.43%, and 21.24 mg/g, 21.05 mg/g, respectively (as shown in Table 2 and Fig. 2b). This may be because the higher the concentration gradient, the greater the drive strength for adsorption, ensuing in a lower potential for sorption and less break-through time ^17^. The percentage removal capacity of the column has declined as the initial dye concentration has improved. Because of the increase in initial concentration, the ICD loading over the given bed depth also increased. The adsorbent sites got blocked over time, and an early breakpoint was initiated with increased concentration ^24^.

### Impact of flow rate on ASAC break-throughplots

**Figure 3:**
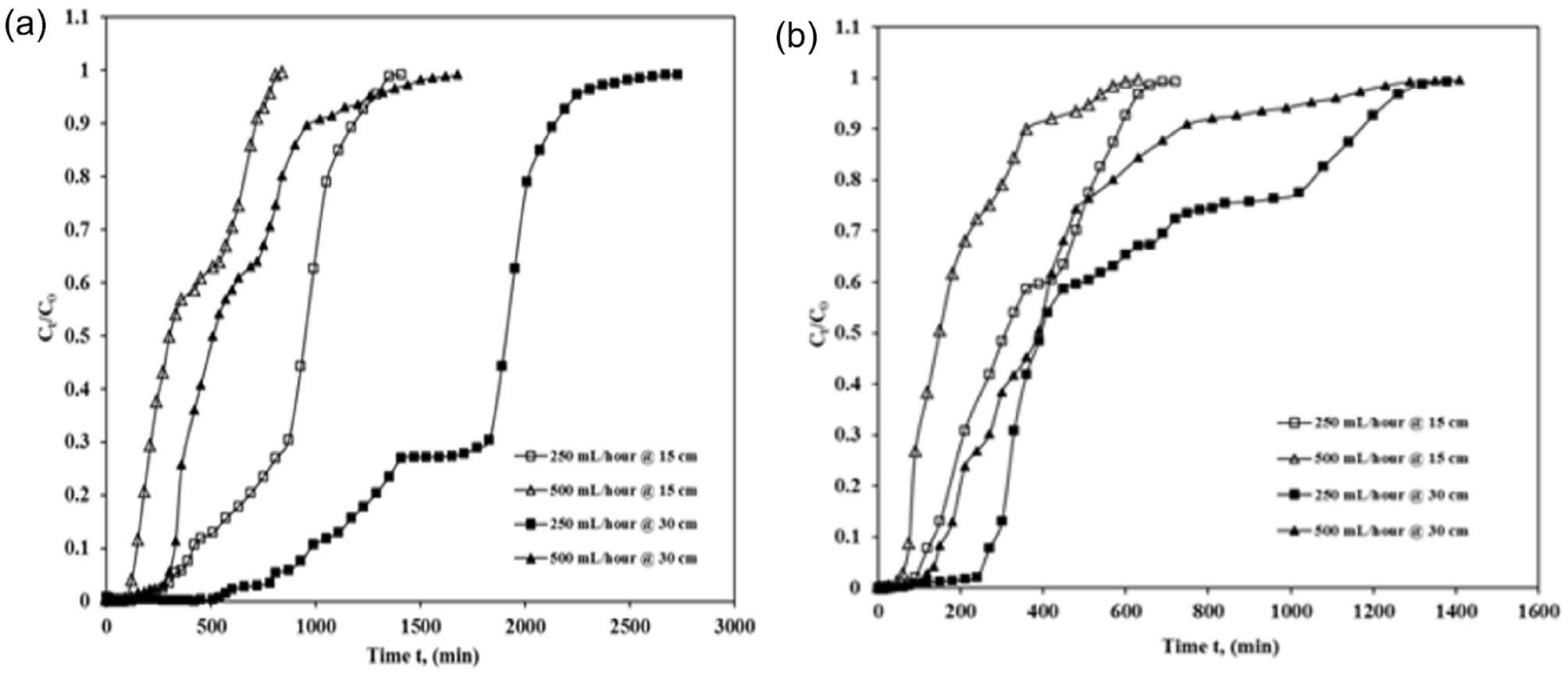
Break-throughplot showing effect of flow rate of ICD sorption by ASAC (z = 15 cm and 30 cm, Q = 250 mL/h and 500 mL/h, at (**a**) C_0_ = 100 mg/L and (**b**) C_0_ = 200 mg/L).

The consequence of flow rate on break-through time for ICD adsorption on fixed ASAC beds was tested at various flow rates of 250 ml/h along with with500 mL/h for100 mg/L and 200 mg/L and column bed depths of 15 cm and 30 cm. Table 3*,* displays the obtained data. Break-through time rises when the flow rate increases and exhaust or saturation time reduces quickly. A higher curve slope shows a substantially reduced in external resistance to mass transfer^24^. For a column bed depth of 15 cm along with the starting ICD concentration of 100 mg/L, the break-throughtime declines from 240 to 120 min, and exhaust time also decreases from 1350 to 810 min when the flow increases from 250 to 500 mL/h (as shown in Fig. 3a). Likewise, in the case of 30 cm of depth, for the starting ICD concentration of 100 mg/L, the break-through time declines from 570 to 270 min, and exhaust time also slows down from 2670 to 1560 min when the flow increases from 250 to 500 mL/h (as shown in Fig. 3a).

**Table 3:** Effects of flow rate on the break-through plots of ASAC.

Column bed depth(cm)	Concentration (mg/L)	Flowrate (mL/h)	Break-through time tb (min)	Exhaust time text (min)	Percentage removal (%)	Adsorption capacity (mg/g)
15	100	250	240	1350	74.48	25.13
		500	120	810	68.38	22.69
30	100	250	570	2670	79.01	24.67
		500	270	1560	75.50	21.24
15	200	250	90	660	57.35	23.43
		500	45	570	47.35	21.99
30	200	250	240	1320	65.79	22.45
		500	120	1290	58.43	21.05

A similar pattern was found in the case of 15 cm of column bed depth and 200 mg/L ICD concentration. The break-through time also decreases from 90 to 45 min, and exhaust time also falls from 660 to 570 min as the flow rises from 250 to 500 mL/h (as shown in Fig. 3b). In the case of 30 cm of column bed depth and 200 mg/L ICD concentration, a related form of the pattern was also observed with the break-through time, which also falls from 240 to 120 minand the exhaust time also decreases from 1320 to 1290 min as the flow grows from 250 to 500 mL/h (as shown in Fig. 3b).

The break-through plot steepens when flow rises, and hence break-through time reduces. This rapid decrease in the breakpoint time for all adsorbents is due to increased flow velocity and a reduction in resident contact time in the adsorption field ^24^. This is because the residence time in the bed is not extended sufficiently for equilibrium to achieve the precise flow rate. Therefore, the interaction time between the solute and adsorbent at higher concentrations is much shorter, consequently lowering the break-through time. It is also observed that when the influent solution’s flow rate rises from 250 to 500 mL/h (as shown in Table 3), ASAC's adsorption capability decreases, and column percentage removal capacity declines because of the inadequate residence time of column’s dye solution ^17^. The solute residence period within the column is a significant parameter for building a packed bed column. Not all of the solute in the solution receives adequate residence time with a high flow rate, resulting in inefficient use of adsorption ability ^25,26^.

### Impact of column bed depth on ASAC break-through plot

**Figure 4:**
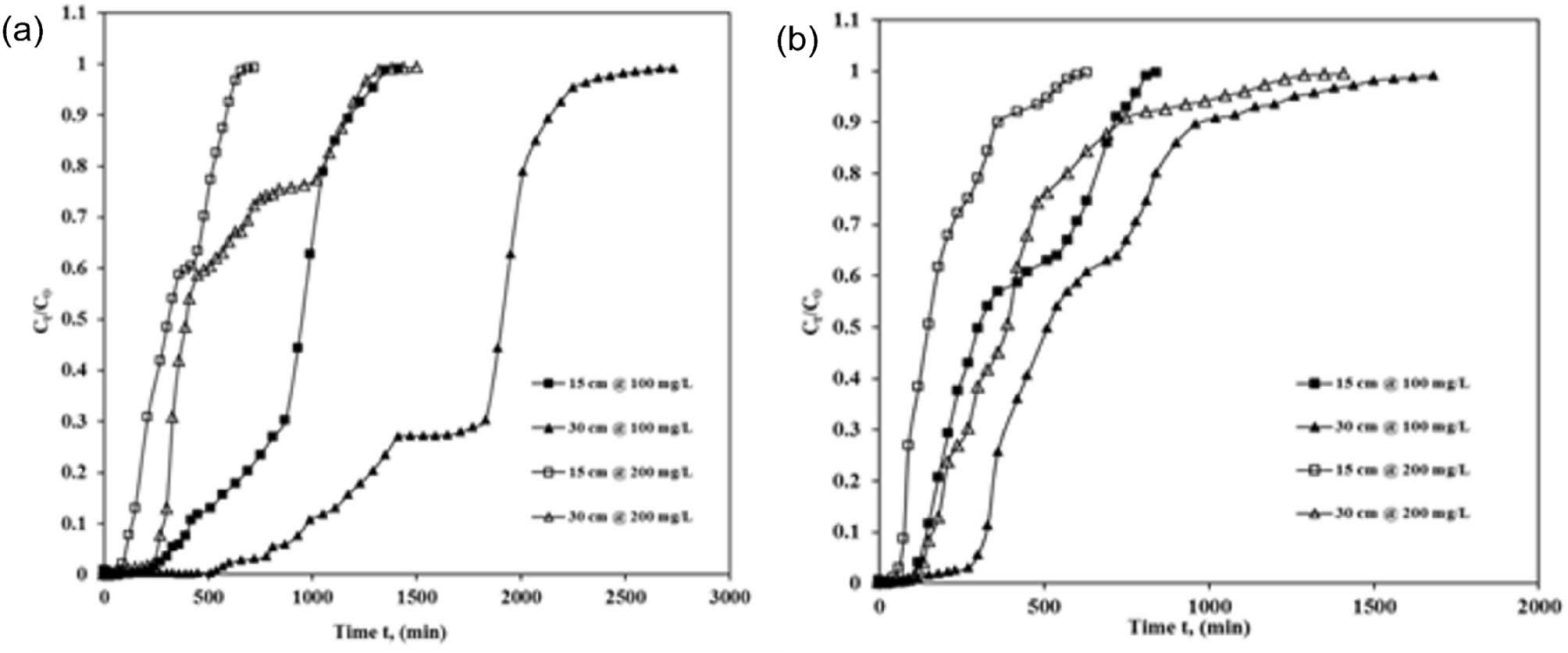
Break-through plot showing effect of bed depth on ICD sorption by ASAC (z = 15 cm and 30 cm,C_0_ = 100 mg/L and 200 mg/L at (**a**) Q = 250 mL/h; (**b**) Q = 500 mL/h).

The sequel of column bed depth on adsorption column parameters was explored by adjusting the bed height of ASAC by 15 cm and 30 cm, using initial ICD concentrations of 100 mg/L and 200 mg/L and flow rates of 250 mL/h and 500 mL/h. The break-through curves and results obtained are shown in Fig. 4 and Table 4.

**Table 4:** Effects of column bed depth on the break-through plot of ASAC.

Concentration (mg/L)	Flowrate (mL/h)	Column bed depth(cm)	Break-through time tb (min)	Exhaust time (min)	Percentage removal (%)	Adsorption capacity (mg/g)
100	250	15	240	1350	74.48	25.13
		30	570	2670	79.01	24.67
200	250	15	90	660	57.35	23.43
		30	240	1320	65.79	22.45
100	500	15	120	810	68.38	22.69
		30	270	1560	75.50	21.24
200	500	15	45	570	47.35	21.99
		30	120	1290	58.43	21.05

It was noticed that both break-through and exhaust time increase with the rise in-depth. In the case of a 250 mL/h flow, the break-through time expands from 240 to 570 min for a first-time ICD concentration of 100 mg/L, and the exhaust time also extends from 1350 to 2670 min. The break-through time expanded from 90 to 240 min for the ICD concentration of 200 mg/L, and exhaust time also increased from 660 to 1320 min bed height extends from 15 to 30 cm, respectively (as shown in Fig. 4a). Similar kind of trends are seen in the case of a flow rate of 500 mL/h; the break-through time for 100 mg/L increased from 120 to 270 min, with exhaust time also rising from 810 to 1560 min, and the break-through time for 200 mg/L increased from 45 to 120 min and the exhaust time also enlarged from 570 to 1290 min as bed height raised from 15 to 30 cm respectively (Fig. 4b).

Bed depth of 15 cm and 30 cm is adopted in the present research work, This Bed depth of 15 cm and 30 cm is predetermined to establish optimum conditions for the sorption performance of ICD onto ASAC. This is because, time of break-through and the time of exhaustion increases with the increasing bed depth (as shown in Table 4). It was observed that at the lowest bed depth, there is no sufficient time for ICD ions to diffuse into the holes of ASAC. The performance of column efficiency also improved with bed height owing to a rise in the measure of adsorbent material that offers more vacancies and newer zones for ICD dye adsorption. But the adsorption capacity of the column decreases with increased bed height, as shown in Table 4.

### Kinetics in column adsorption study and Error analysis

To scrutinize the performance in the uninterrupted adsorption phase of ICD onto ASAC, three kinetic plots, i.e., Bed-depth-service-time, Yoon–Nelson and Thomas plots, and the error analysis by least-square of errors system were measured obtained for the ICD sorption onto ASAC.

#### Thomas modelling of break-throughplots

The Thomas model defines and calculates break-through plots and column efficiency. This model has been extracted from the premise that the drive strength has reverse action kinetics and undertakes Langmuir's desorption/adsorption and insignificant radial/axial scattering kinetics^27^. The Thomas plot measuring various concentrations is given in (Eq. 1.)

1 $$\frac{{C_{t} }}{C0} = \frac{1}{{1 + \exp \left( {\frac{{k_{T} q_{0} m}}{Q} - kTC0t} \right)}}$$

Thomas’ equation’s linear form is given as (Eq. 2)

2 $$In\left( {\frac{{C_{0} }}{Ct} - 1} \right) = \frac{{k_{T} q_{0} m}}{Q} - kTC0t$$

**Figure 5:**
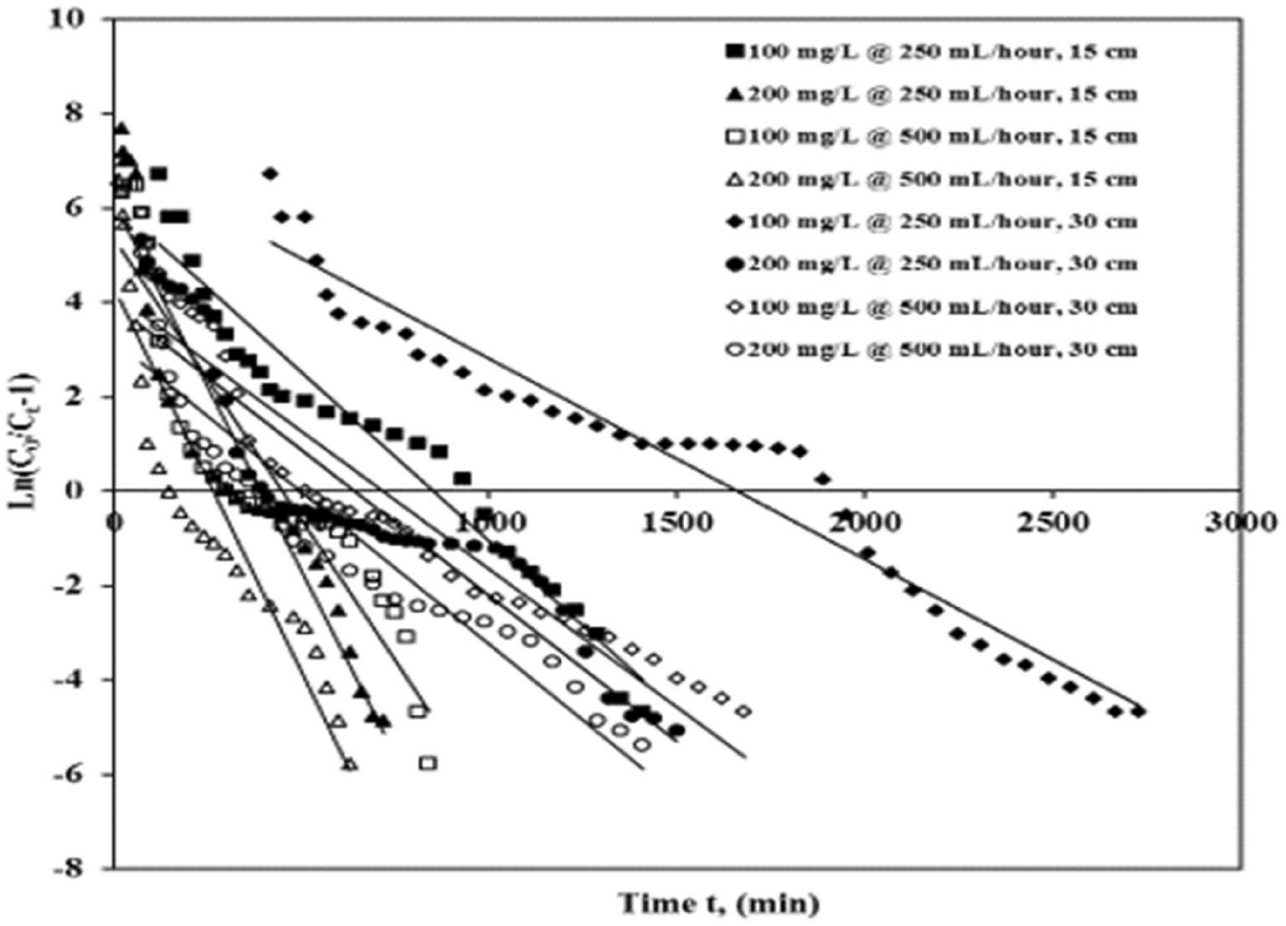
Thomas break-through plot of ICD sorption by ASAC (z = 15 cm and 30 cm, C_0_ = 100 mg/L and 200 mg/L, Q = 250 mL/h and 500 mL/h).

*C*_0_ is the concentration of influent ICD (mg/L), C_*t*_ is the concentration of effluent ICD (mg/L), *k*_*T*_ is the Thomas rate constant (L/mg/min), q_0_ is the equilibrium ICD uptake per gram of ASAC (mg/g), Q is the flow rate (mL/h), m is the ASAC volume (g), and *t* is time (min). A linear plot of ln [(C_0_/C_*t*_) − 1] against time *t* was used to measure *k*_*T*_ and q_*0*_ values from the intersection and slope. Table 5 and Fig. 5 show the model’s parameters as well as the correlation coefficient.

**Table 5:** Parameters of Thomas plot for ICD sorption onto ASAC.

Column bed depth z (cm)	Concentration (mg/L)	Flow rate (mL/h)	Break through time tb (min)	*k*_*T*_ (L/mg/min)	q_*total*_ (mg)	q_*exp*_ (mg/g)	q_*calc*_ (mg/g)	R^2^	S.S
15	100	250	240	0.00006957	366.07	25.13	25.02	0.949	3.97 E−03
15	200		90	0.00007488	282.51	23.43	23.42	0.911	1.85 E−02
30	100	250	570	0.00004273	718.72	24.67	23.71	0.953	1.35 E−02
30	200		240	0.00003095	478.41	22.45	18.37	0.860	3.19 E−02
15	100	500	120	0.00011827	329.45	22.69	25.97	0.860	2.64 E−02
15	200		45	0.00007987	319.75	21.99	30.82	0.852	3.50 E−02
30	100	500	270	0.00005764	500.19	21.24	20.81	0.910	1.63 E−02
30	200		120	0.00003195	439.30	21.05	28.69	0.888	1.52 E−02

The investigational column statistics were applied to the Thomas model equation to obtain (*k*_*T*_), i.e., Thomas rate constant and (q_*0*_) maximum adsorption capacity. The relative parameters and regression coefficient (R^2^) were determined using a correlation basin. The results of relative parameters and values of error analysis by the least-square of errors method (S.S) (that is less than or up to 0.004) are also listed in Table 5. The correlation coefficient for regression was between 0.852 and 0.953, so there is a substantial correlation between *t* and C_*t*_/C_*0*_.

Table 5 depicts that with the rise from 100 to 200 mg/L of ICD concentrations, the value of q_*exp*_ decreases while the Thomas rate constant *k*_*T*_ increases. This may be because of the original concentration contrast between the solution dye and the adsorbent dye and the driving force for adsorbent adsorption^21^. Therefore, the higher drive strength to achieve a lower ICD concentration results in better column motion. Similarly, as the flow rate expanded from 250 to 500 mL/h, the rate of q_*exp*_ decreased while the rate of k_T_ increased. The rate of q_*exp*_ improved noticeably as the depth (z) amplified from 15 to 30 cm, while the rate of k_T_ decreased. Thus, lesser influent ICD concentration, lesser flow rate, and greater or higher depth will improve the efficiency of the column for ICD adsorption on ASAC. Thomas rate constant and contrast of adsorption capacity values acquired from experimental data and calculations showed that they were significantly close for given situations that specify the Thomas model's applicability.

#### Yoon–Nelson modelling of break-throughplots

The Yoon–Nelson plot^28^ was worn to learn the break-through action of ICD adsorption on ASAC. It takes into account the hypothesis that a decrease in the sorption probability rate is relative to adsorbate sorption and adsorbate break-through probability^28^.

The Yoon–Nelson kinetic plot for the column is given as (Eq. 3):

3 $$\frac{{C_{t} }}{C0} = \frac{1}{{1 + \exp \;[k_{YN} (\tau - t)]}}$$

The linear plotof one component structure is presented as(Eq. 4):

4 $$In\left( {\frac{Ct}{{C_{0} - Ct}}} \right) = k_{YN} t - \tau k_{YN}$$

As *C*_*0*_ is the influent ICD concentration (mg/L), *C*_*t*_ is the effluent ICD concentration (mg/L), *k*_*YN*_ is the Yoon–Nelson rate constant (L/min), is the time needed for 50% adsorbate break-through (min), along with *t* is the sampling time (min).

**Figure 6:**
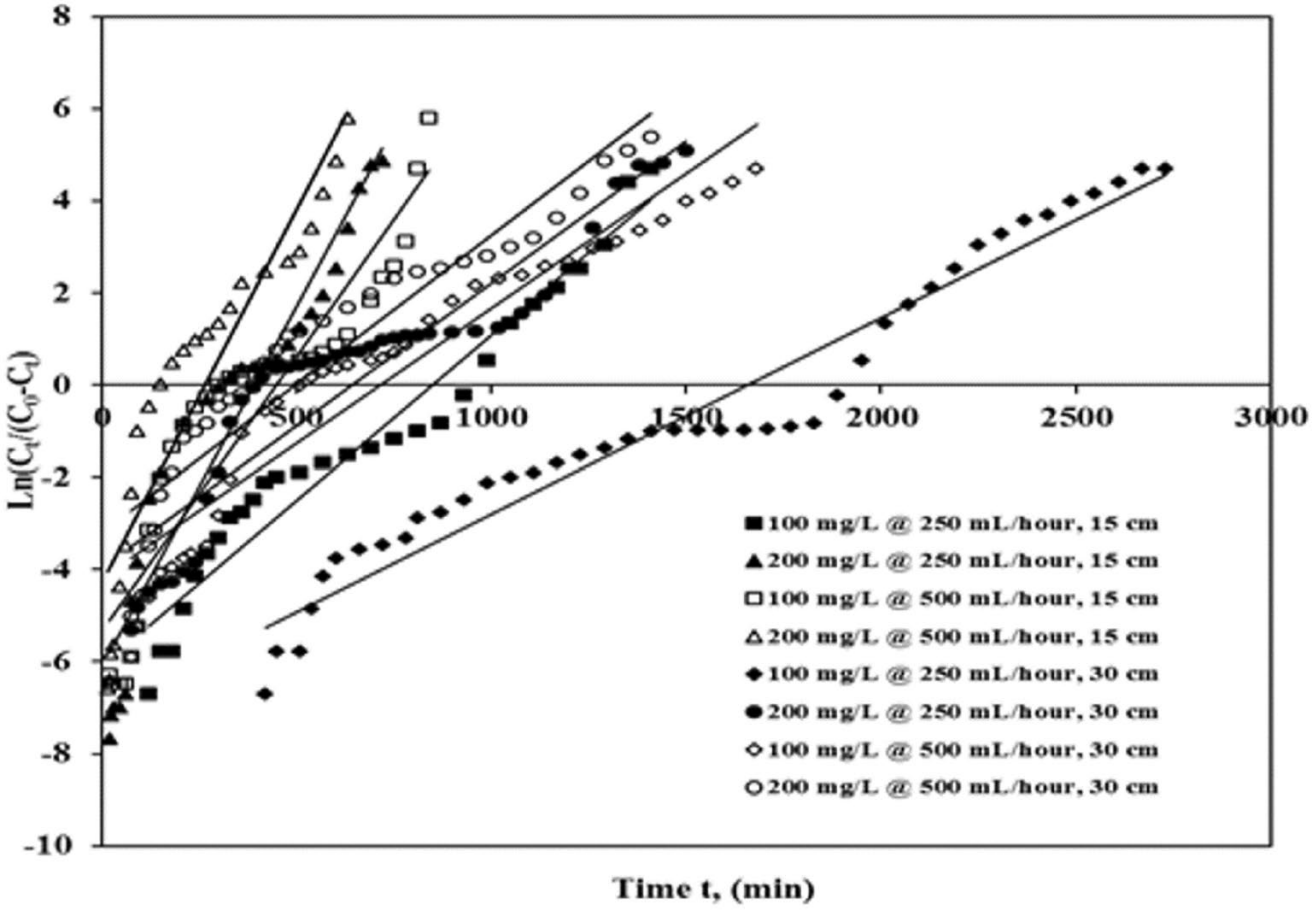
Yoon–Nelson break-through plot of ICD sorption by ASAC (z = 15 cm and 30 cm, *C*_*0*_ = 100 mg/L and 200 mg/L, Q = 250 mL/h and 500 mL/h).

A linearized design of *ln [C*_*t*_*/(C*_*0*_* − Ct)]* against sample time (*t*) was worn to compute*k*_*YN*_ along with the slope and intercept. In Table 6 and Fig. 6, the parameters of the model and the coefficients of correlation are presented.

**Table 6:** Parameters of Yoon–Nelson plot for ICD sorption onto ASAC.

Column bed depth, z (cm)	Concentration (mg/L)	Flow rate (mL/h)	Break-through time, tb (min)	q_*total*_ (mg)	q_*exp*_ (mg/g)	q_*calc*_ (mg/g)	*k*_*YN*_ (L/min)	τ (min)	R^2^	S.S
15	100	250	240	366.07	25.13	25.02	0.0070	869.43	0.949	4.27 E−03
15	200		90	282.51	23.43	23.42	0.0150	408.07	0.911	2.09 E−02
30	100	250	570	718.72	24.67	23.71	0.0043	1639.81	0.953	1.57 E−02
30	200		240	478.41	22.45	18.37	0.0062	640.29	0.860	3.46 E−02
15	100	500	120	329.45	22.69	25.97	0.0119	449.85	0.860	2.95 E−02
15	200		45	319.75	21.99	30.82	0.0160	268.44	0.852	4.00 E−02
30	100	500	270	500.19	21.24	20.81	0.0058	720.93	0.910	1.77 E−02
30	200		120	439.30	21.05	28.69	0.0064	499.70	0.888	1.62 E−02

When a basic hypothetical Yoon–Nelson model was applied to ASAC (Table 6) to understand the break-throughbehaviour of ICD by obtaining the values of constant time *τ* and *k*_*YN*_, the Yoon–Nelson rate constant *k*_*YN*_ improved along with 50% adsorbate break-through time (*τ*) falls with the grows in influent ICD concentration and flow rate for a given column bed depth. With the bed volume, i.e., flow rate increasing from 250 to 500 mL/h, the value has expanded, while the standard of *k*_*YN*_ has reduced. Table 6 also shows that the break-through time decreases for a given column depth as the flow rate (Q) increases from 250 to 500 mL/h, and the influent ICD concentration (C_0_) increases from 100 to 200 mg/L. In all cases, the regression correlation coefficient R^2^ was found to be nearly close to unity.

The total quantity of adsorbate (q_*total*_) decreases as the influent ICD concentration and flow rate increase. Though all the parameters obtained in a model satisfy the Yoon–Nelson hypothesis, an increase in* k*_*YN*_ values with a decrease in τ values and slightly higher S.S values compared to other models may slightly deviate (negligible) internal significance of this model to a marginal extent.

#### Bed-depth-service-time analysis (BDST) modelling of break-through plots

The hypothesis of the BDST plot depicts that the pace of sorption is synchronized by the reaction between adsorbate (ICD) and adsorbent (ASAC)^29^. BDST is a common researchplot for forecasting the connection between depth and time concerning concentrations and various sorption characteristics. The BDST plot is indicated as (Eq. 5):

5 $$\frac{{C_{t} }}{C0} = \frac{1}{{1 + \exp \left( {\frac{{k_{a} N_{0} Z}}{u} - k_{a} C0t} \right)}}$$

The adsorption capacity by the BDST model is expressed as (Eq. 6):

6 $$q_{0} = \frac{{N_{0} ZQ}}{um}$$

*C*_*0*_ is the inflow ICD concentration (mg/L), *C*_*t*_ is the outflow ICD concentration (mg/L), *k*_*a*_is the BDST model rate constant (L/mg/min), *N*_*0*_ is the saturation concentration (mg/L), z is the bed depth (centimetre), and *u* is the influent linear velocity (centimetre/min), where *t*is the sampling time (min) *q*_0_ represents the equilibrium ICD uptake per gram of adsorbent (mg/g), Q means the flow rate (mL/h).

**Figure 7:**
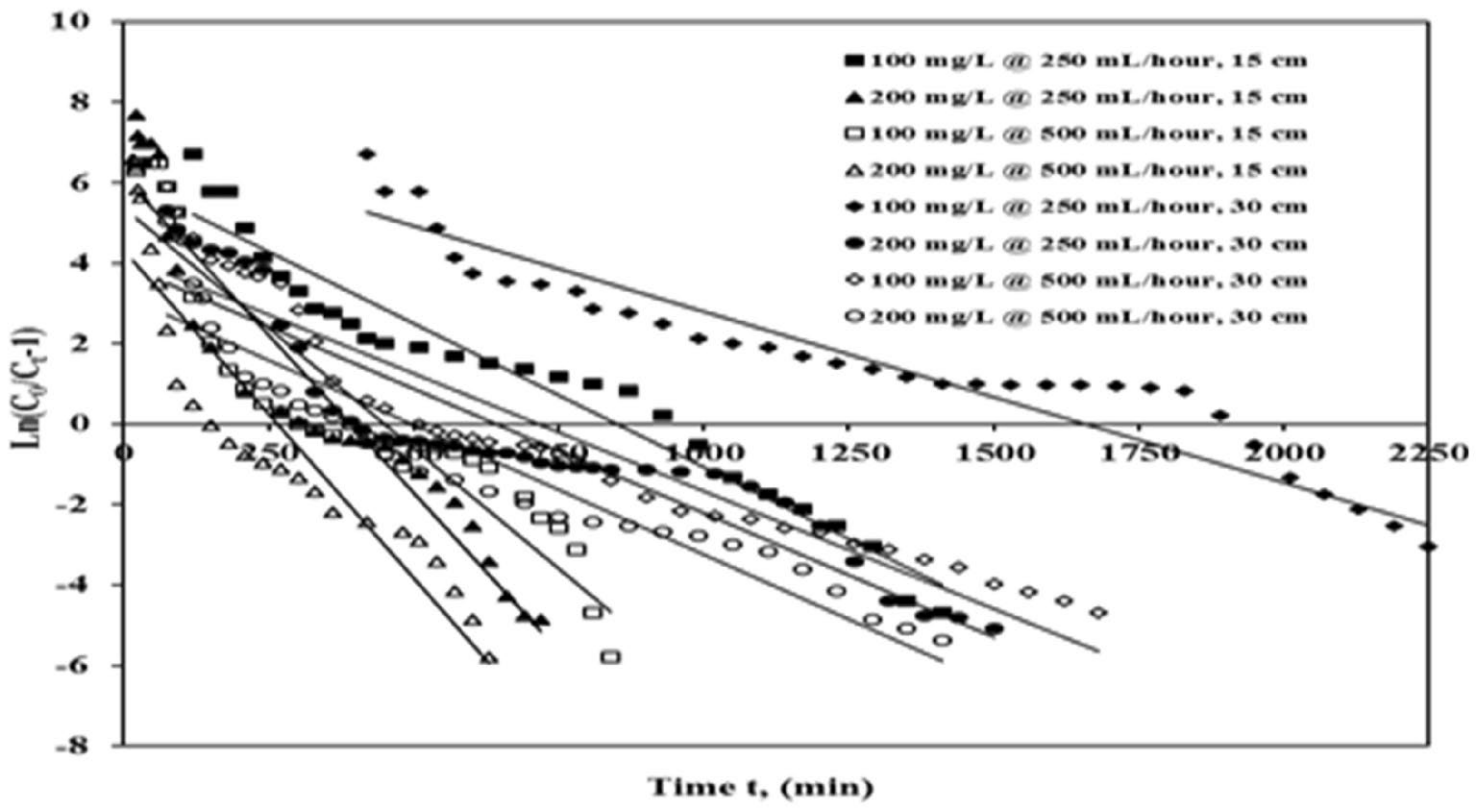
BDSTbreak-through plot of ICD sorption by ASAC (z = 15 cm and 30 cm, *C*_*0*_ = 100 mg/L and 200 mg/L, Q = 250 mL/h and 500 mL/h).

Also, m represents the volume of adsorbent (ASAC) in the column (g). As *C*_*t*_*/C*_*0*_ is plotted against sampling time (*t*) and column bed width, a straight line is obtained (z). The BDST model measures the adsorption power, saturation concentration, and BDST rate constant (*k*_*a*_). The model parameters, total adsorbed quantity (*q*_*total*_) and the correlation coefficients are presented in Table 7 and Fig. 7.

**Table 7:** Parameter of BDST plot for ICD sorption on ASAC.

Column bed depth z (cm)	Concentration (mg/L)	Flow rate (mL/h)	Break through time tb (min)	q_*total*_ (mg)	q_*exp*_ (mg/g)	q_*calc*_ (mg/g)	*k*_*a*_ (L/mg/min)	*N*_*0*_ (mg/L)	R^2^	S.S
15	100	250	240	366.07	25.13	25.02	6.95719E−05	29,159.29	0.949	4.11 E-−3
15	200		90	282.51	23.43	23.42	7.48848E−05	27,246.29	0.911	1.92 E−02
30	100	250	570	718.72	24.67	23.71	4.2737E−05	54,996.83	0.953	1.45 E−02
30	200		240	478.41	22.45	18.37	3.09524E−05	42,751.69	0.860	3.27 E−02
15	100	500	120	329.45	22.69	25.97	1.18272E−04	30,174.46	0.860	2.74 E−02
15	200		45	319.75	21.99	30.82	7.98771E−05	35,846.73	0.852	3.65 E−02
30	100	500	270	500.19	21.24	20.81	5.76453E−05	48,357.83	0.910	1.68 E−02
30	200		120	439.30	21.05	28.69	3.19508E−05	66,729.58	0.888	1.57E−02

The result (Table 7; Fig. 7) shows that break-through time reduces with the rise in influent ICD concentration and flow rate for a given depth of column bed. While the *k*_*a*_ and *N*_*0*_ values increase with the same. The BDST model variables will continue to advance the methods for different flow rates and other influential dye concentrations without additional laboratory experiments. To measure the column output at new feasible flow rates and influent dye concentrations, the BDST equation was used at either flow rate or influent ICD concentration. The error analysis values obtained by the least square method (S.S) were smaller than those obtained by the Yoon–Nelson, and robust predictions were found for adjusting feed concentration and flow rate. In every one ofthe cases, the regression coefficient R^2^ was approximately equal to one, indicating the significance andrationality of the BDST model for the current system.

Columns with a wide range of possible flow rates and concentrations can be generated using the model and measured constants.These outcomes show that the optimized conditions can be utilized to estimate adsorption efficiency on ASAC under desirable operating conditions for the ICD adsorption process.

#### Error analysis

As diverse equations are considered to calculate linear regression correlation coefficients (R^2^) values, these values may significantly influence the accuracy throughout the linear regression examination; subsequently, the nonlinear regression investigation can be a superior alternative in evading such errors. As a result, the parameters of various kinetic models were determined using nonlinear correlation coefficients examination and the least square of errors procedure ^17^. Error analysis was conducted to validate which model gives better results. The relative formula for error analysis by least-square of errors method (S.S) is provided by the following equation (Eq. 7) and respectively shown in Tables 5, 6, and 7 for Thomas, Yoon–Nelson, and BDST model

7 $$S.S. = \frac{{\sum {\left[ {\left( {C_{t} /C_{0} } \right)_{c} - \left( {C_{t} /C_{0} } \right)_{e} } \right]^{2} } }}{N}$$

Here (C_t_/C_0_)_c_ is the ratio of effluent to influent ICD concentrations calculated using Thomas, Yoon–Nelson and BDST plots, and (C_t_/C_0_)_e_ is the ratio of effluent to influent ICD concentrations calculated using experimentation conditions^30,31^. N is the number of experimental data points. It is important to evaluate the data using S.S according to the coefficients (R^2^) criteria to validate the most suitable and best-fitting kinetic model.

**Figure 8:**
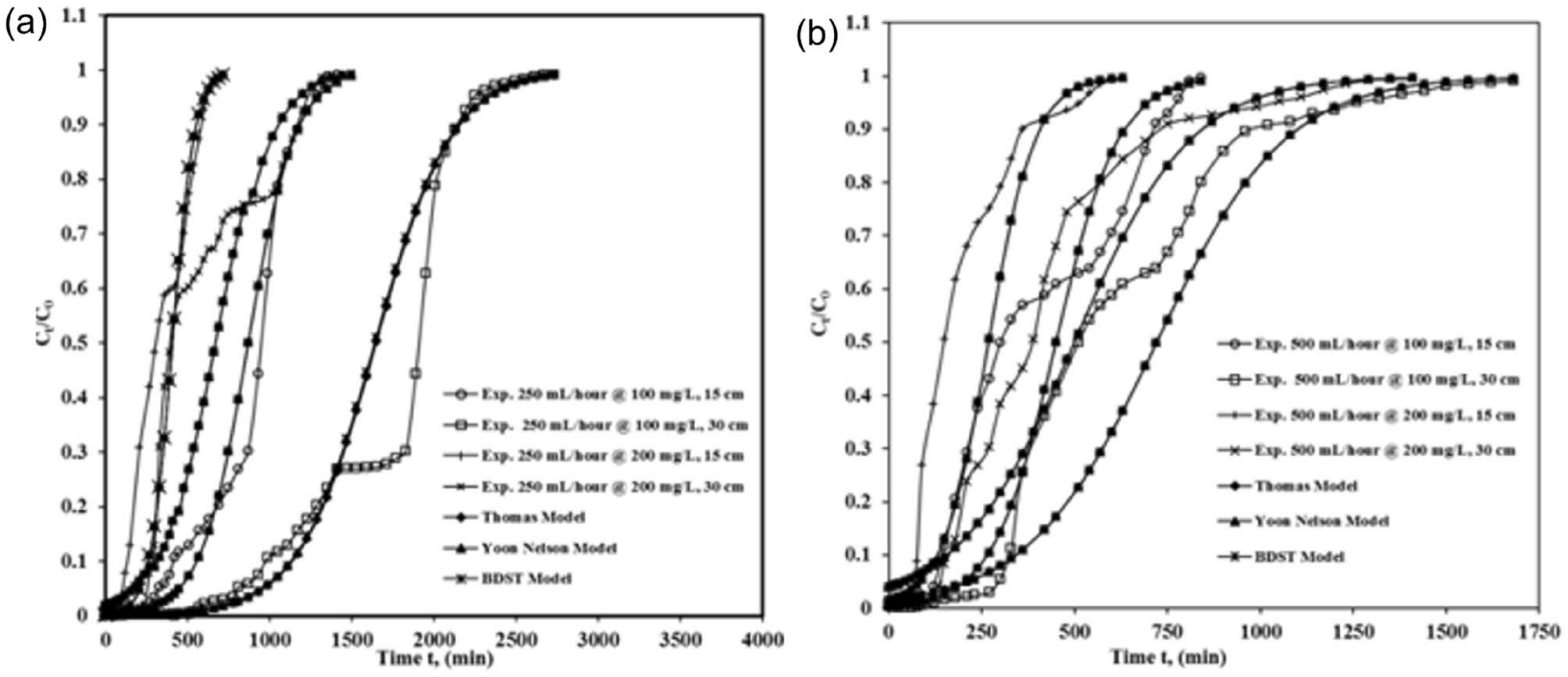
Thomas, Yoon–Nelson and BDST models profiles.

Figure 8 shows profiles measured and projected by Thomas, Yoon–Nelson and BDST models. It shows experimental break-throughplots comparison of ICDonto ASAC for column bed depth z = 15 cm and 30 cm, flow rate Q = 250 mL/h (Fig. 8a) and 500 mL/h (Fig. 8b), influent ICD concentration C_0_ = 100 mg/L and 200 mg/L).

Figure 8 break-throughplot comparison of ICD onto ASAC (z = 15 cm and 30 cm, C_0_ = 100 mg/L and 200 mg/L at (a) Q = 250 mL/h (b) Q = 500 mL/h). It was clear from the figures (Fig. 8) that the contract between the experimental points and the estimated normalized ICD concentrations was significantly strong. All three models were suitable for adsorption processes in continuous fixed-bed adsorption systems where internal and external diffusions are not the limiting and restricting stage^21,32^. The column kinetic study revealed that the experimental break-through plots compared satisfactorily with the break-through profiles calculated by Thomas, Yoon–Nelson, and BDST models. The correlation coefficients showed that every one ofthe threemodels matches the experimental results well and is equivalent to one another. But if comparing the values of factors, constants, and error analysis obtained by the least square method (S.S) for all the three models, it could be considered that BDST best followed the Thomas model along with the Yoon–Nelson models in relating the development of ICD adsorption onto ASAC.

### B–E–T surface area and B–E–T pore volume

B–E–T surface area and B–E–T pore volume of ASAC were analyzed using Brunauer, Emmett and Teller (B–E–T) method by ASTMD-3663-03. The standard test was conducted using ASAP 2020 (Micrometrics, USA) surface area and porosity analyzer. The *B–E–T* surface area and *B–E–T* pore volume of raw sawdust were 543.28 m^2^/g and 0.1925 cm^3^/g respectively. At the same time, *B–E–T* surface area and *B–E–T* pore volume of ASAC were obtained to be 737.76 m^2^/g and 0.2583 cm^3^/g, respectively. This rise in *B–E–T* surface area and *B–E–T* pore volume is due to the physico-chemical activation of raw sawdust into activated carbon (ASAC).

### S-E-M, F-T-I-R and X-R-D analysis

**Figure 9:**
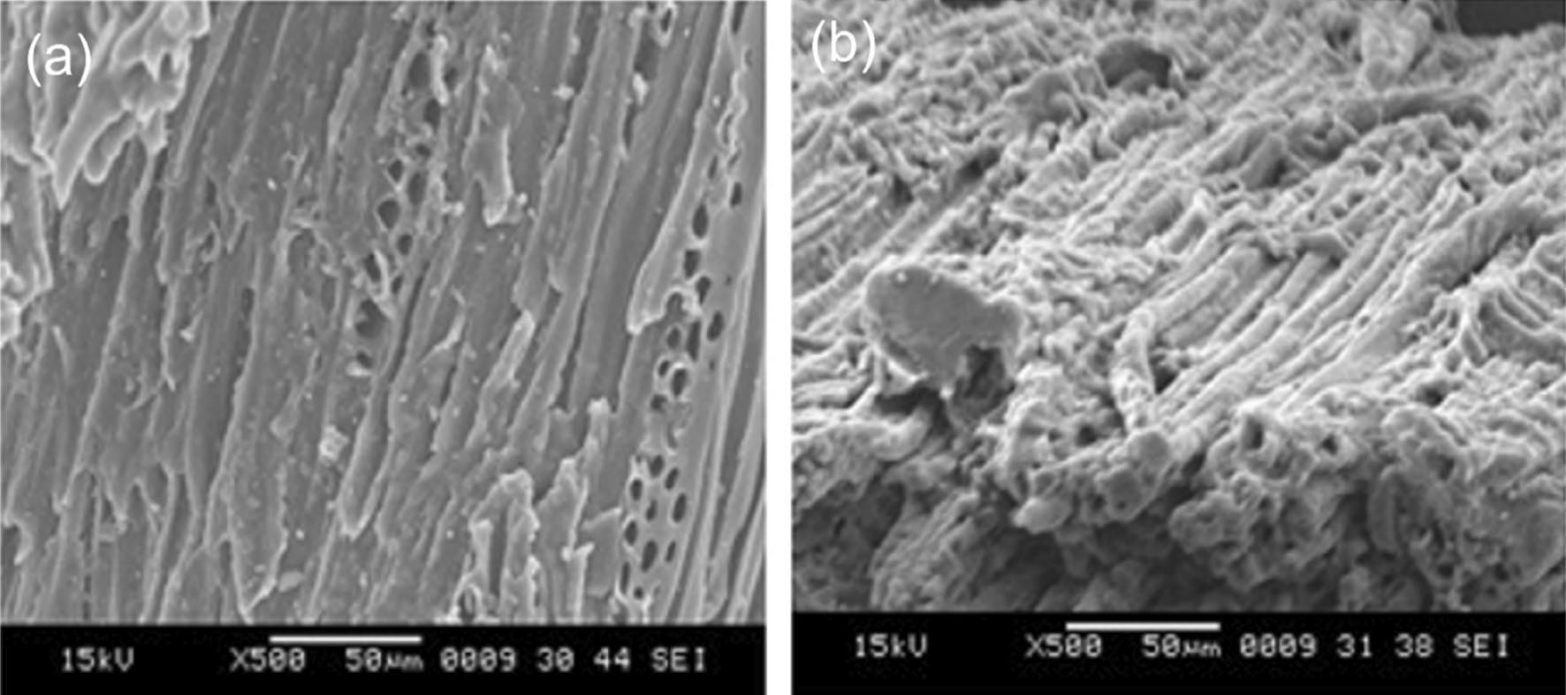
S-E-M pictures of (**a**) raw ASAC and (**b**) ICD laden ASAC.

S-E-M analysis explains the surface morphology and porosity of ASAC. ASAC laden with ICD was tested at 15 kV, 500× magnifications using a scanning electron microscope. From the picture of raw and laden ASAC (Fig. 9), it was noticed that the nature of ASAC is micro-porous. Such micro-pores are answerable for the biosorption of ICD onto the surface of ASAC. Such porosity is responsible for entering ICD molecules to penetrate the ling hemicellulose composition. They intermingle with the ASAC surface and diverse functional bonds ^11,17^.

**Figure 10:**
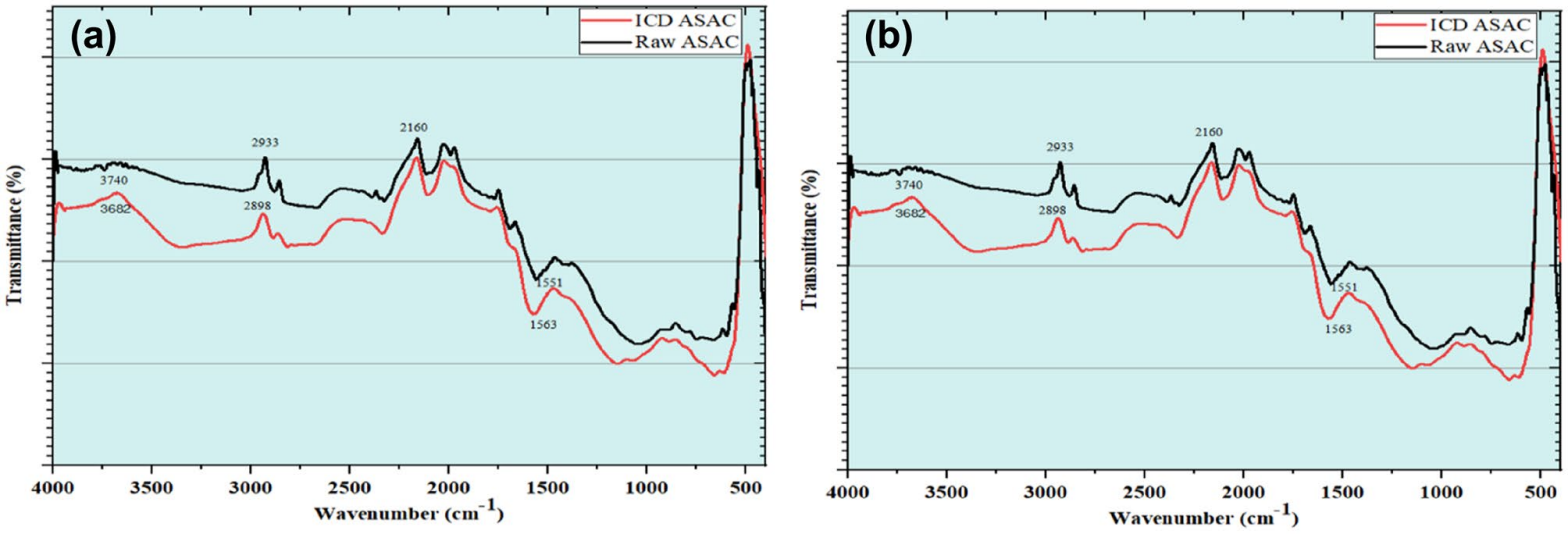
F-T-I-Rspectroscope of raw ASAC and ICD laden ASAC.

The F-T-I-R spectroscopes are usually worn-out to recognize the distinguishing functional assemblies with excellent sorption capability. F-T-I-R spectroscopy monitors the chemistry of the ASAC surface and ICD-ASAC surface ^11,17^. F-T-I-R spectroscopes of raw ASAC and ICD-laden ASAC are depicted in Fig. 10. This spectroscope shows the occurrence of distinguishing functional assemblies onto ASAC. The summit stretched in raw ASAC at 3670.06 cm^−1^ because of the –OH assembly, which vaguely shifts to 3682.03 cm^−1^ after ICD sorption onto ASAC. The group stretched in raw ASAC at 2933 cm^−1^ depicts well-built C–H links that swing faintly in ICD-laden ASAC to 2898 cm^−1^. The summit stretched in raw ASAC at 2160 cm^−1^ describes the occurrence of a weak C–C linkage thatdoes not swing after ICD adsorption. Similarly, the summit at 1551 cm^−1^ for raw ASAC swings to 1563 cm^−1^ for ICD laded ASAC^33^. This depicts the strong occurrence of C–OH and C–O groups. This swinging of summits validates the sorption of ICD onto ASAC.

**Figure 11:**
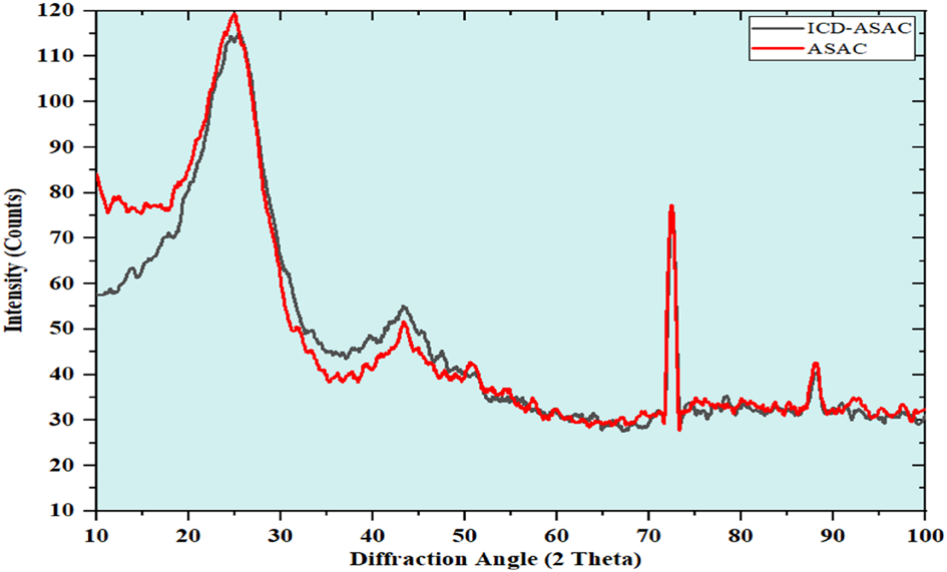
X-R-D spectrum of raw ASAC and ICD laden ASAC.

X-R-D technique is a tool to analyze the crystalline or amorphous constitution of the adsorbents. The adsorption process may lead to changes in the adsorbent's constitution. Hence, understanding the molecular constitution and crystalline/amorphous constitution of the ASAC would provide valuable information regarding adsorption. Figure 11 shows the X-R-D spectra of raw ASAC and ICD-laden ASAC^34–36^. From Fig. 11, before the adsorption of ICD onto ASAC, the surface of ASAC showed hollow peaks, which indicated its amorphous nature. After adsorption with ICD, a reduction in the porous structure of the ASAC can be observed.

## Conclusion

The present study shows the utilization of ASAC as an effective solution for removing ICD from wastewater. A continuous study of the fixed bed adsorption column on ASAC for the treatment of ICD discovered that ASAC could be used as an adsorbent material in industrial wastewater treatment to remove dyes from the solution. The adsorption of ICD for a given column bed depth was influenced by the flow rate and the ICD concentration. It was found that break-through and exhaust time occurred faster for shallower bed depths (15 cm) and increased gradually as column depth increased (30 cm). It was also observed that with an increase in initial dye solution concentration (from 100 to 200 mg/L) and flow rate (from 250 to 500 mL/h), break-through time and exhaust time decreased. The percentage removal efficiency and adsorption capacity of ASAC increase for lower initial ICD concentration and flow rate. The column study revealed that the maximum removal efficiency of ICD and adsorption capacity of ASAC was found to be about 47.35% and 21.99 mg/g, respectively, at lower column depth (15 cm), which increased respectively to 79.01% and 24.67 mg/g at higher column depth (30 cm), with the initial dye concentration reduced from 200 to 100 mg/L, allowing flow rate to reduce from 500 to 250 mL/h. The column kinetic study and error analysis also depicted that the experimental break-through plots compared satisfactorily with the break-through profiles calculated by Thomas, Yoon–Nelson and BDST models. Through comparing the values of variables, constants, and error analysis for all three models in relating the mechanism of ICD adsorption onto ASAC, it was also discovered that Thomas was better approached over BDST and Yoon–Nelson models. The comparison of correlation coefficients shows that all three models match the experimental results well and are equal in fixed-bed adsorption systems. The *B–E–T* surface area and *B–E–T* pore volume of raw sawdust were 543.28 m^2^/g and 0.1925 cm^3^/g respectively. Whereas *B–E–T* surface area and *B–E–T* pore volume of ASAC were found to be 737.76 m^2^/g and 0.2583 cm^3^/g, respectively. This rise in *B–E–T* surface area and *B–E–T* pore volume is due to physico-chemical activation of raw sawdust into activated carbon (ASAC). *S-E-M* analysis and *X-R-D* analysis reveal the micro-porous and amorphous nature of ASAC. *F-T-I-R* spectroscopes indicate distinctive functional assemblies like –OH group, C–H bond, C–C bond, C–OH, and C–O groups on ASAC.It could be computed that the *Acacia nilotica* sawdust activated column can be considered as an alternative treatment option for industrial (specifically dye bearing) wastewater.

## Data Availability

The datasets used and analysed during the current study are available from the corresponding author on reasonable request.
